# HOMELETTE: a unified interface to homology modelling software

**DOI:** 10.1093/bioinformatics/btab866

**Published:** 2021-12-25

**Authors:** Philipp Junk, Christina Kiel

**Affiliations:** Systems Biology Ireland and UCD Charles Institute of Dermatology, School of Medicine, University College Dublin, Dublin 4, Ireland

## Abstract

**Summary:**

Homology modelling, the technique of generating models of 3D protein structures based on experimental structures from related proteins, has become increasingly popular over the years. An abundance of different tools for model generation and model evaluation is available from various research groups. We present HOMELETTE, an interface which implements a unified programmatic access to these tools. This allows for the assemble of custom pipelines from pre- or self-implemented building blocks.

**Availability and implementation:**

HOMELETTE is implemented in Python, compatible with version 3.6 and newer. It is distributed under the MIT license. Documentation and tutorials are available at Read the Docs (https://homelette.readthedocs.io/). The latest version of HOMELETTE is available on PyPI (https://pypi.org/project/homelette/) and GitHub (https://github.com/PhilippJunk/homelette). A full installation of the latest version of HOMELETTE with all dependencies is also available as a Docker container (https://hub.docker.com/r/philippjunk/homelette_template).

**Supplementary information:**

Supplementary data are available at *Bioinformatics* online.

## 1 Introduction

Access to homology modelling tools has become increasingly simpler over the last years. There is a multitude of web services such as SWISS-MODEL offering total automation of the whole process. These are great tools for small homology modelling projects (Waterhouse *et al.*, 2018). However, medium to large scale projects, aiming to model the structures of tens or hundreds of proteins with different homology modelling software in a full- or semi-automated manner are faced with a very tedious exercise. Most of the popular homology modelling services offer command line tools. However, these tools come with different interfaces and work with different file types. The same is true for software aiming to evaluate homology models.

**Fig. 1.**
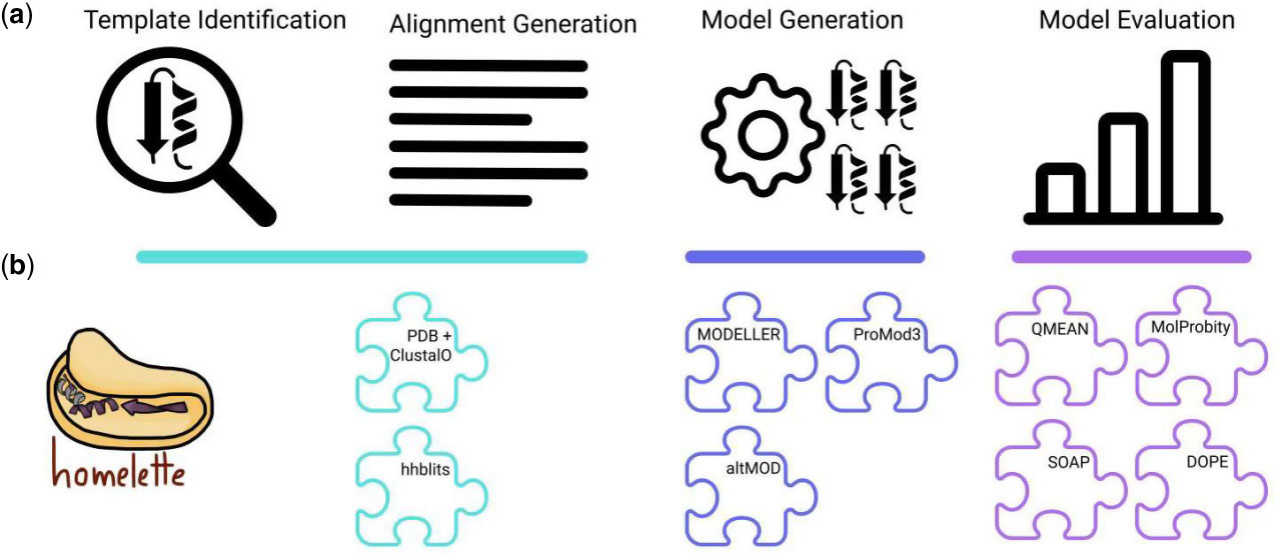
Homology modelling pipeline. (**a**) General pipeline of homology modelling from left to right. (**b**) Building blocks implemented in HOMELETTE and how they correspond to the steps in homology modelling

The general flow of a homology modelling pipeline is depicted in Figure  1a (Webb and Sali, 2016). Usual requirements for most homology modelling software are a multiple sequence alignment (MSA) of the target sequence against one or multiple template sequences, as well as template structures. Using the information from the alignment and the template structure(s), a homology modelling algorithm assembles one or multiple models. Afterwards, these are evaluated by some evaluation metrics in order to select the best model(s).

Exchanging components of the pipeline such as the modelling algorithm or the evaluation metrics is not trivial due to the problems outlined above. Therefore, the motivation behind HOMELETTE is to provide a modular homology modelling interface that can be used to construct pipelines with diverse modelling and evaluation tools within the same interface. The focus is also on making it easy for the user to implement new building blocks that fit into the framework. This interface can be used to easily assemble custom pipelines and streamline medium to large scale homology modelling projects (Fig.  1b).

## 2 Implementation

The HOMELETTE interface is fully implemented in Python. Python is a popular and accessible programming language extensively used in the scientific community (Van Rossum and Drake, 2009).

HOMELETTE is built with modular design principles in mind. Template identification/alignment generation, model generation and model evaluation are designed as interchangeable building blocks that interact with the other components of the pipeline in an identical manner. This allows for the easy assembly of custom pipelines by freely combining these building blocks. Alignment generation and template processing building blocks are available for identifying templates with the RCSB Search Web API using MMseq2 (Rose *et al.*, 2021; Steinegger and Söding, 2017) and align them with Clustal Omega (Sievers *et al.*, 2011; Sievers and Higgins, 2018), or using HHSuite3 (Steinegger *et al.*, 2019). Model generation building blocks are currently available for MODELLER (Sali and Blundell, 1993; Webb and Sali, 2016), altMOD (Janson *et al.*, 2019) and ProMod3 (Biasini *et al.*, 2013; Studer *et al.*, 2021). Model evaluation building blocks are available for DOPE scores (Shen and Sali, 2006), SOAP scores (Dong *et al.*, 2013), QMEAN (Benkert *et al.*, 2008; Benkert *et al.*, 2011), QMEAN DisCo (Studer *et al.*, 2020) and MolProbity (Chen *et al.*, 2010, Williams *et al.*, 2018). A good model is expected to have a low DOPE score, a low SOAP score, a high QMEAN score and a MolProbity score as close to 0 as possible. A list of the implemented building blocks is available in Supplementary Table S1.

In addition, new building blocks can be implemented and seamlessly fit into existing pipelines allowing for even further customization. This is particularly useful for integrating software for which no building block is available yet into the framework. Users are strongly encouraged to share their custom building blocks with the community, and an extension framework has been set up to make this possible.

Extensive documentation and tutorials teach the user how to use these building blocks, how to implement new building blocks and how to assemble them into more complex pipelines. The documentation is available online at https://homelette.readthedocs.io/. The tutorials are hosted together with the documentation, or as interactive Jupyter notebooks on the GitHub page and in the Docker container.

HOMELETTE does not have any model building or model evaluating capacities on its own, but its strength comes from the integration of different software. Due to these design choices, it is reliant on third-party software (Supplementary Table S1). All currently integrated software is freely available for academic research. The documentation gives instruction on how to acquire and install third-party software. Alternatively, HOMELETTE is also available as a Docker container with all third-party software already installed.

## 3 Application

As an example for the custom assembly of alignment generating, homology modelling and model evaluation building blocks into custom pipelines, the ARAF protein was modelled (Supplementary Fig. S1). Starting from the sequence, the templates 3NY5 (BRAF) and 4G0N (RAF1) were identified, aligned and processed. In order to show how different modelling building blocks can be used interchangeably, two MODELLER building blocks with different parameters for model refinement were used. Evaluation was performed by using SOAP scores and MolProbity scores, which were summarized to a combined score using Borda count (Supplementary Fig. S1b). As expected, the modelling routine that spends more time on model refinement generates better models. There are also differences between the templates to be observed. The code to execute this example as well as to generate the visualization is made fully available in Tutorial 7.

## 4 Conclusion

There are three major determinants for the quality of a homology model. These are the alignment used, the quality of the template structures and the algorithm chosen for generating the models (Webb and Sali, 2016). HOMELETTE leaves the selection of all three determinants in the hand of the user. The user has agency which modelling software to use and compare, as well as full control over generating and refining the alignment and selecting templates.

We explain and demonstrate the use of HOMELETTE in the series of eight tutorials. The tutorials culminate in a tutorial about pipeline assembly, which has been shown as an example pipeline for a proof of concept in this publication (Supplementary Fig. S1).

In conclusion, HOMELETTE offers a unified, simple and well-documented interface to a multitude of popular homology model and model evaluation software. Its modular design principles allow users to assemble their own pipelines in an easy and consistent manner. Simple implementation and extensive documentation make it possible to extend HOMELETTE with other software, while retaining the same programmatic interface. This gives users even more freedom to assemble the best custom pipeline for their particular project. This could prove useful for large scale projects such as the structural modelling of whole biological systems.

## Supplementary Material

btab866_Supplementary_Data
