# Correction to: HOMELETTE: a unified interface to homology modelling software

**DOI:** 10.1093/bioinformatics/btad086

**Published:** 2023-02-17

This is a correction to: Philipp Junk, Christina Kiel, HOMELETTE: a unified interface to homology modelling software, Bioinformatics, Volume 38, Issue 6, March 2022, Pages 1749–1751, https://doi.org/10.1093/bioinformatics/btab866

The originally published version of this manuscript included an incorrect supplementary data file.

The Publisher apologizes for this error, which has now been corrected.

## Supplementary Material

btad086_Supplementary_Data

