# Supplementary material for: HOMELETTE: a unified interface to homology modelling software
Source: Bioinformatics. 2021 Dec 25;38(6):1749–51. doi: 10.1093/bioinformatics/btab866 (PMC8896651; doi:10.1093/bioinformatics/btab866)
Supplement: btab866_Supplementary_Data [file btab866_supplementary_data.docx]

**

Figure S1:** (a) Example of a modelling pipeline. For the query sequence (ARAF RBD in this case), templates were identified using the RCSB Search API, and an alignment was generated using Clustal Omega. 3NY5 and 4G0N were selected as templates. Models were generated using the MODELLER homology modelling software with two different parameter sets. The models were evaluated using SOAP scores and MolProbity scores. (b) The combined scores of the models generated by the pipeline described in (a). Scores were combined by using Borda count. A higher score indicates a better model. The full code of this example is available as Tutorial 7 (<https://homelette.readthedocs.io/en/latest/Tutorial7_AssemblingPipelines.html>).

**Table S1**:

| Building Block | Type | Program/Software | Reference |
| --- | --- | --- | --- |
| AlignmentGenerator_pdb | Alignment generation, Template processing | RCSB Search API (MMseq2), Clustal Omega | Rose *et al.*, 2021; Steinegger and Söding, 2017; Sievers *et al.*, 2011; Sievers and Higgins, 2018 |
| AlignmentGenerator_hhblits | Alignment generation, Template processing | HHSuite3 | Steinegger *et al.*, 2019 |
| AlignmentGenerator_from_aln | Alignment generation, Template processing | - | - |
| Routine_automodel_default | Model generation | MODELLER | Sali and Blundell, 1993; Webb and Sali, 2016 |
| Routine_automodel_slow | Model generation | MODELLER | Sali and Blundell, 1993; Webb and Sali, 2016 |
| Routine_altmod_default | Model generation | altMOD | Janson *et al.*, 2009 |
| Routine_altmod_slow | Model generation | altMOD | Janson *et al.*, 2009 |
| Routine_promod3 | Model generation | ProMod3 | Biasini *et al.*, 2013; Studer *et al.*, 2021 |
| Routine_complex_automodel_default | Model generation | MODELLER | Sali and Blundell, 1993; Webb and Sali, 2016 |
| Routine_complex_automodel_slow | Model generation | MODELLER | Sali and Blundell, 1993; Webb and Sali, 2016 |
| Routine_complex_altmod_default | Model generation | altMOD | Janson *et al.*, 2009 |
| Routine_complex_altmod_slow | Model generation | altMOD | Janson *et al.*, 2009 |
| Routine_loopmodel_default | Model generation | MODELLER | Sali and Blundell, 1993; Webb and Sali, 2016 |
| Routine_loopmodel_slow | Model generation | MODELLER | Sali and Blundell, 1993; Webb and Sali, 2016 |
| Evaluation_dope | Model evaluation | MODELLER | Shen and Sali, 2006 |
| Evaluation_soap_protein | Model evaluation | MODELLER | Dong *et al.*, 2013 |
| Evaluation_soap_pp | Model evaluation | MODELLER | Dong *et al.*, 2013 |
| Evaluation_qmean4 | Model evaluation | QMEAN | Benkert *et al.*, 2008; 2016 |
| Evaluation_qmean6 | Model evaluation | QMEAN | Benkert *et al.*, 2008; 2016 |
| Evaluation_qmeandisco | Model evaluation | QMEAN | Benkert *et al.*, 2008; 2016; Studer *et al.*, 2020 |
| Evaluation_mol_probity | Model evaluation | MolProbity | Chen *et al.*, 2010; Williams *et al.*, 2018 |

**References**

Benkert, P. *et al.* (2011) Toward the estimation of the absolute quality of individual protein structure models. *Bioinformatics*, 27(3), pp.343–350.

Benkert, P. *et al.* (2008) QMEAN: A comprehensive scoring function for model quality assessment. *Proteins: Structure, Function and Genetics*, 71(1), pp.261–277.

Biasini, M. *et al.* (2013) OpenStructure: An integrated software framework for computational structural biology. *Acta Crystallographica Section D: Biological Crystallography*, 69(5), pp.701–709.

Chen, V.B. *et al.* (2010) MolProbity: All-atom structure validation for macromolecular crystallography. *Acta Crystallographica Section D: Biological Crystallography*, 66(1), pp.12–21.

Dong, G.Q. *et al.* (2013) Optimized atomic statistical potentials: Assessment of protein interfaces and loops. *Bioinformatics*, 29(24), pp.3158–3166.

Janson G. *et al.* (2019) Revisiting the “satisfaction of spatial restraints” approach of MODELLER for protein homology modeling. *PLoS Comput Biol* 15(12): e1007219.

Rose, Y. *et al.* (2021) RCSB Protein Data Bank: Architectural Advances Towards Integrated Searching and Efficient Access to Macromolecular Structure Data from the PDB Archive. *Journal of Molecular Biology*, 433(11), p.166704.

Sali, A. and Blundell, T.L., (1993) Comparative protein modelling by satisfaction of spatial restraints. *Journal of Molecular Biology*, 234(3), pp.779–815.

Shen, M. and Sali, A., (2006) Statistical potential for assessment and prediction of protein structures. *Protein Science*, 15(11), pp.2507–2524.

Sievers, F. and Higgins, D.G., (2018) Clustal Omega for making accurate alignments of many protein sequences. *Protein Science*, 27(1), pp.135–145.

Sievers, F. *et al.* (2011) Fast, scalable generation of high-quality protein multiple sequence alignments using Clustal Omega. *Molecular Systems Biology*, 7(1), p.539.

Steinegger, M. *et al.* (2019) HH-suite3 for fast remote homology detection and deep protein annotation. *BMC Bioinformatics*, 20(1), pp.1–15.

Steinegger, M. and Söding, J., (2017) MMseqs2 enables sensitive protein sequence searching for the analysis of massive data sets. *Nature Biotechnology* 2017 35:11, 35(11), pp.1026–1028.

Studer, G. *et al.* (2020) QMEANDisCo-distance constraints applied on model quality estimation. *Bioinformatics*, 36(6), pp.1765–1771.

Studer, G. *et al.* (2021) ProMod3—A versatile homology modelling toolbox. PLOS Computational Biology, 17(1), p.e1008667.

Webb, B. and Sali, A., (2016) Comparative Protein Structure Modeling Using MODELLER. *Current Protocols in Bioinformatics*, 54(1), pp.5.6.1-5.6.37.

Williams, C.J. *et al.* 2018. MolProbity: More and better reference data for improved all-atom structure validation. *Protein Science*, 27(1), pp.293–315.
